# Supplementary material for: Waste not, want not: revisiting the analysis that called into question the practice of rarefaction
Source: mSphere. 2023 Dec 6;9(1):e00355-23. doi: 10.1128/msphere.00355-23 (PMC10826360; doi:10.1128/msphere.00355-23)
Supplement: Supplemental Materials — Supplemental text and figures. [file msphere.00355-23-s0001.pdf]

Supplementary text

Quotes from selected papers that cited WNWN and conflated “rarefying”/“rarefy” and “rarefaction”. Many of these papers also attributed “rarefaction” to WNWN. Rarefying, rarefy, and rarefaction are bolded to make the words easier to identify. All references have been updated to reflect the numbering in the current study. Citations to WNWN are indicated by (1) and all numbering is in reference to the list of references in this Supplement. Quotes are ordered chronologically.

- “Additionally, **rarefaction** has recently been shown to introduce errors in analyses, and alternatives to rarefaction have been proposed (1)” - Quoted from (2). Implies that rarefaction and WNWN’s rarefying are the same thing.
- “**Rarefaction** is analytically problematic and poses multiple statistical problems: (i) omission of available valid data, (ii) the estimation of overdispersion is more difficult due to data loss, (iii) loss of power (type II error), (iv) dependence on an arbitrary threshold and (v) additional uncertainty due to the randomness in **rarefaction** (1).” - Quoted from (3). Implies that rarefaction and WNWN’s rarefying are the same thing.
- “McMurdie and Holmes (1) penalized the **rarefying** technique for dropping the lowest fifteenth percentile of sample library sizes in their simulations by counting the dropped samples as”incorrectly clustered.” Because the 15th percentile was used to set **rarefaction** depth, this capped clustering accuracy at 85%.” - Quoted from (4). An example of a paper from the QIIME development community using rarefaction and rarefying interchangeably.
- “**Rarefaction** has a limited ability in this regard since the total sum constraint still exists after **rarefaction**. In addition, it suffers from a great power loss due to the discard of a large number of reads (1).” - Quoted from (5). Implies that rarefaction and WNWN’s rarefying are the same thing.
- “Another popular normalization approach is **rarefaction**, which consists on subsampling the same number of reads for each sample so that all samples have the same number of total counts. **Rarefaction** is not recommended because it entails the loss of important information (1).” - Quoted from (6). Implies that rarefaction and WNWN’s rarefying are the same thing.
- “Unfortunately, **rarefaction** is neither justifiable nor necessary, a view framed statistically by McMurdie and Holmes (1) in the context of comparison of relative abundances.” - Quoted from (7). Implies that rarefaction and WNWN’s rarefying are the same thing.

- “Some studies perform **rarefaction** to adjust for differences in library size due to unexhaustive metagenomic sampling. Although several pipelines provide this functionality, it has been found inadmissible for metagenomics microbiome studies as it discards many reads leading to decreased sensitivity in differential abundance testing (1) and biased estimates for alpha diversity (7).” - Quoted from (8). Implies that rarefaction and WNWN’s rarefying are the same thing.

- “Another widespread practice is to **rarefy** the count data to force the samples to have the same number of total sequence reads (9), at the expense of discarding vast amounts of information (1).” - Quoted from (10). Hughes and Hellmann (9) describe traditional rarefaction with multiple subsamplings.

- “Note that the terms **rarefying** and **rarefaction** are used interchangeably in microbiome literature (1). **Rarefying** was first recommended for microbiome data to deal with rare taxa (11), which impact some measures of alpha and beta diversities (1).” - Quoted from (12). As the quote indicates they use rarefying and rarefaction interchangeably although the cited WNWN indicates that they are not the same. In addition, the first review article to advocate the use of rarefaction was Hughes et al. (13) and examples of its use can be found earlier (14, 15);

- “Ways to tackle this include total library size normalization and **rarefaction**, with both remaining debated to date (1, 4).” - Quoted from (16). Implies that rarefaction and WNWN’s rarefying are the same thing.

- “**Rarefaction** is a widely used normalization technique that involves the random subsampling of sequences from the initial sample library to a selected normalized library size. This process is often dismissed as statistically invalid because subsampling effectively discards a portion of the observed sequences, yet it remains prevalent in practice and the suitability of **rarefying**, relative to many other normalization approaches, for diversity analysis has been argued” - Quoted by (17). Uses rarefaction and rarefying interchangeably. This paper describes a new method as “repeated rarefying”, which is the traditional rarefaction approach.

- “Confronted with technical variation as well as the overall increase in raw sequencing data generated per sample over the years, **rarefaction** (or downsampling) was suggested to standardize within and across dataset comparisons . . . However, sequencing depth-based downsizing procedures were soon criticized, not only for being wasteful and discarding information on low-abundant taxa (1), but also for being unsuited when applied to communities characterized by substantial variation in cell density (18).” - Quoted by (19). Implies that rarefaction and WNWN’s rarefying are the same thing.

- “Some have suggested that data should be **rarefied** (4, 20) to a common sampling depth, typically to the level of the sample with fewest sequences, while others argue that such **rarefaction** is ‘inadmissible’ and favor approaches that transform or scale sequence counts (1, 21).” - Quoted by (22). Uses rarefied and rarefaction interchangeably and implies that both are the same as WNWN’s rarefying.

- “**Rarefaction** has been criticized for wasting data since we effectively remove a portion of the data in the downsampling procedure (1).” - Quoted by (23). Here and elsewhere in this paper the authors use rarefaction and rarefying interchangeably and implies that both are the same as WNWN’s rarefying.

- “**Rarefying** (also referred to as **rarefaction**) is a popular but widely criticized technique for correcting uneven sequencing depths. It involves randomly discarding counts in samples until all samples have the same predefined number of total counts.” - Quoted by (24). As the quote indicates they use rarefying and rarefaction interchangeably although the cited WNWN indicates that they are not the same.

- “One commonly used method is to **rarefy** the data; that is, ASVs or OTUs within a sample are randomly subsampled without replacement to a preselected depth that is the same across all samples. The outcome of this is that all samples will have the same number of ASVs and any samples with fewer ASVs than the **rarefaction** level will be removed from the dataset. The level for **rarefaction** can be decided using a **rarefaction** curve, a method in which each sample is subsampled at multiple levels (e.g. 1,000 reads, 2,000 reads, 3,000 reads. . .), and the number of unique features or another metric of individual sample diversity of each sample at each level is measured and plotted. When the plot begins to level off after an initial climb up, the corresponding number of sequences indicates an appropriate sampling depth. The appropriate number to **rarefy** must then be balanced with the number of samples that may be dropped from the dataset which do not meet that minimum. An advantage of **rarefaction** is that it may be a more appropriate measure of very low-abundance (”rare”) ASVs. This can in turn increase the accuracy of the data, as low biomass samples often have contamination and quality concerns (25). There are also disadvantages to this method, the most obvious of which is the discarding of valuable data. Clearly, this is less than ideal as the researcher must pay for the samples and sequences, and in cases where the samples are very valuable or difficult to obtain the loss of data may be destructive to the overall experimental integrity. Additionally, the loss of statistical power by removing sequences from a sample could lead to a loss of differences between two samples (1). The statistical consequences extend beyond this, as **rarefying**

equalizes sample variance by adding artificial uncertainty (1).” - Quoted by (26). The authors use rarefaction and rarefying interchangeably and implies that both are the same as WNWN’s rarefying.

References

1. **McMurdie PJ**, **Holmes S**. 2014. Waste not, want not: Why rarefying microbiome data is inadmissible. PLoS Computational Biology **10**:e1003531. doi:10.1371/journal.pcbi.1003531.

2. **Goodrich JK**, **Rienzi SCD**, **Poole AC**, **Koren O**, **Walters WA**, **Caporaso JG**, **Knight R**, **Ley RE**. 2014. Conducting a microbiome study. Cell **158**:250–262. doi:10.1016/j.cell.2014.06.037.

3. **Bálint M**, **Bahram M**, **Eren AM**, **Faust K**, **Fuhrman JA**, **Lindahl B**, **OHara RB**, **Öpik M**, **Sogin ML**, **Unterseher M**, **Tedersoo L**. 2016. Millions of reads, thousands of taxa: Microbial community structure and associations analyzed via marker genes. FEMS Microbiology Reviews **40**:686–700. doi:10.1093/femsre/fuw017.

4. **Weiss S**, **Xu ZZ**, **Peddada S**, **Amir A**, **Bittinger K**, **Gonzalez A**, **Lozupone C**, **Zaneveld JR**, **Vázquez-Baeza Y**, **Birmingham A**, **Hyde ER**, **Knight R**. 2017. Normalization and microbial differential abundance strategies depend upon data characteristics. Microbiome **5**:27. doi:10.1186/s40168-017-0237-y.

5. **Chen L**, **Reeve J**, **Zhang L**, **Huang S**, **Wang X**, **Chen J**. 2018. GMPR: A robust normalization method for zero-inflated count data with application to microbiome sequencing data. PeerJ **6**:e4600. doi:10.7717/peerj.4600.

6. **Calle ML**. 2019. Statistical analysis of metagenomics data. Genomics & Informatics **17**:e6. doi:10.5808/gi.2019.17.1.e6.

7. **Willis AD**. 2019. Rarefaction, alpha diversity, and statistics. Frontiers in Microbiology **10**:2407. doi:10.3389/fmicb.2019.02407.

8. **Eetemadi A**, **Rai N**, **Pereira BMP**, **Kim M**, **Schmitz H**, **Tagkopoulos I**. 2020. The computational diet: A review of computational methods across diet, microbiome, and health. Frontiers in Microbiology **11**:393. doi:10.3389/fmicb.2020.00393.

9. **Hughes JB**, **Hellmann JJ**. 2005. The application of rarefaction techniques to molecular inventories of microbial diversity, p. 292–308. *In* Methods in enzymology. Elsevier.

10. **Leite MFA**, **Kuramae EE**. 2020. You must choose, but choose wisely: Model-based approaches for microbial community analysis. Soil Biology and Biochemistry **151**:108042. doi:10.1016/j.soilbio.2020.108042.

11. **Lozupone C**, **Lladser ME**, **Knights D**, **Stombaugh J**, **Knight R**. 2010. UniFrac: An effective distance metric for microbial community comparison. The ISME Journal **5**:169–172. doi:10.1038/ismej.2010.133.

12. **Lin H**, **Peddada SD**. 2020. Analysis of microbial compositions: A review of normalization and differential abundance analysis. npj Biofilms and Microbiomes **6**:60. doi:10.1038/s41522-020-00160-w.

13. **Hughes JB**, **Hellmann JJ**, **Ricketts TH**, **Bohannan BJM**. 2001. Counting the uncountable: Statistical approaches to estimating microbial diversity. Applied and Environmental Microbiology **67**:4399–4406. doi:10.1128/aem.67.10.4399-4406.2001.

14. **Dunbar J**, **Takala S**, **Barns SM**, **Davis JA**, **Kuske CR**. 1999. Levels of bacterial community diversity in four arid soils compared by cultivation and 16S rRNA gene cloning. Applied and Environmental Microbiology **65**:1662–1669. doi:10.1128/aem.65.4.1662-1669.1999.

15. **Moyer CL**, **Tiedje JM**, **Dobbs FC**, **Karl DM**. 1998. Diversity of deep-sea hydrothermal vent archaea from loihi seamount, hawaii. Deep Sea Research Part II: Topical Studies in Oceanography **45**:303–317. doi:10.1016/s0967-0645(97)00081-7.

16. **Alteio LV**, **Séneca J**, **Canarini A**, **Angel R**, **Jansa J**, **Guseva K**, **Kaiser C**, **Richter A**, **Schmidt H**. 2021. A critical perspective on interpreting amplicon sequencing data in soil ecological research. Soil Biology and Biochemistry **160**:108357. doi:10.1016/j.soilbio.2021.108357.

17. **Cameron ES**, **Schmidt PJ**, **Tremblay BJ-M**, **Emelko MB**, **Müller KM**. 2021. Enhancing diversity analysis by repeatedly rarefying next generation sequencing data describing microbial communities. Scientific Reports **11**:22302. doi:10.1038/s41598-021-01636-1.

18. **Vandeputte D**, **Kathagen G**, **D'hoe K**, **Vieira-Silva S**, **Valles-Colomer M**, **Sabino J**, **Wang J**, **Tito RY**, **Commer LD**, **Darzi Y**, **Vermeire S**, **Falony G**, **Raes J**. 2017. Quantitative microbiome profiling links gut community variation to microbial load. Nature **551**:507–511. doi:10.1038/nature24460.

19. **Lloréns-Rico V**, **Vieira-Silva S**, **Gonçalves PJ**, **Falony G**, **Raes J**. 2021. Benchmarking microbiome transformations favors experimental quantitative approaches to address compositionality and sampling depth biases. Nature Communications **12**:3562. doi:10.1038/s41467-021-23821-6.

20. **McKnight DT**, **Huerlimann R**, **Bower DS**, **Schwarzkopf L**, **Alford RA**, **Zenger KR**. 2018. Methods for normalizing microbiome data: An ecological perspective. Methods in Ecology and Evolution **10**:389–400. doi:10.1111/2041-210x.13115.

21. **Gloor GB**, **Macklaim JM**, **Pawlowsky-Glahn V**, **Egozcue JJ**. 2017. Microbiome datasets are compositional: And this is not optional. Frontiers in Microbiology **8**:2224. doi:10.3389/fmicb.2017.02224.

22. **Neu AT**, **Allen EE**, **Roy K**. 2021. Defining and quantifying the core microbiome: Challenges and prospects. Proceedings of the National Academy of Sciences **118**:e2104429118. doi:10.1073/pnas.2104429118.

23. **Hong J**, **Karaoz U**, **Valpine P de**, **Fithian W**. 2022. To rarefy or not to rarefy: Robustness and efficiency trade-offs of rarefying microbiome data. Bioinformatics **38**:2389–2396. doi:10.1093/bioinformatics/btac127.

24. **Swift D**, **Cresswell K**, **Johnson R**, **Stilianoudakis S**, **Wei X**. 2022. A review of normalization and differential abundance methods for microbiome counts data. WIREs Computational Statistics **15**:e1586. doi:10.1002/wics.1586.

25. **Kennedy K**, **Hall MW**, **Lynch MDJ**, **Moreno-Hagelsieb G**, **Neufeld JD**. 2014. Evaluating bias of Illumina-based bacterial 16S rRNA gene profiles. Applied and Environmental Microbiology **80**:5717–5722. doi:10.1128/aem.01451-14.

26. **Weinroth MD**, **Belk AD**, **Dean C**, **Noyes N**, **Dittoe DK**, **Rothrock MJ**, **Ricke SC**, **Myer PR**, **Henniger MT**, **Ramírez GA**, **Oakley BB**, **Summers KL**, **Miles AM**, **Ault-Seay TB**, **Yu Z**, **Metcalf JL**, **Wells JE**. 2022. Considerations and best practices in animal science 16S ribosomal RNA gene sequencing microbiome studies. Journal of Animal Science **100**:skab346. doi:10.1093/jas/skab346.

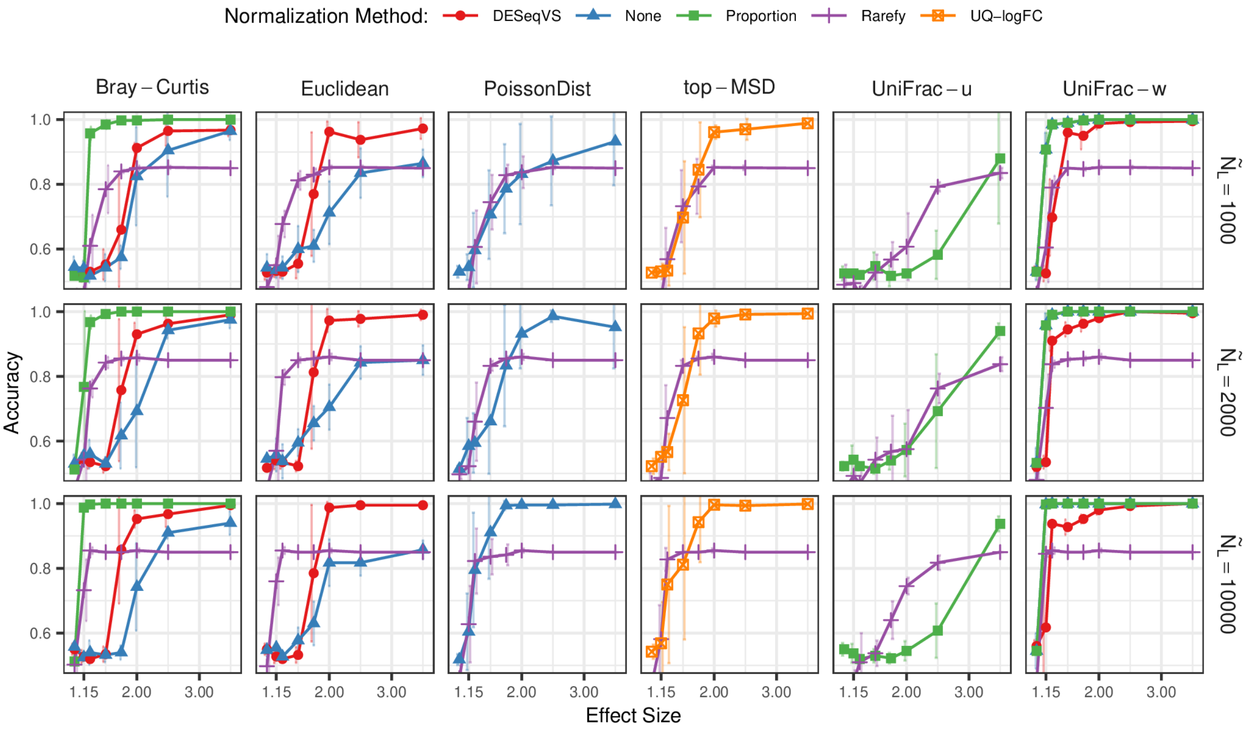

Figure S1. Re-running the R markdown files provided in Protocol S1 of WNWN qualitatively reproduced Figure 4 from WNWN.

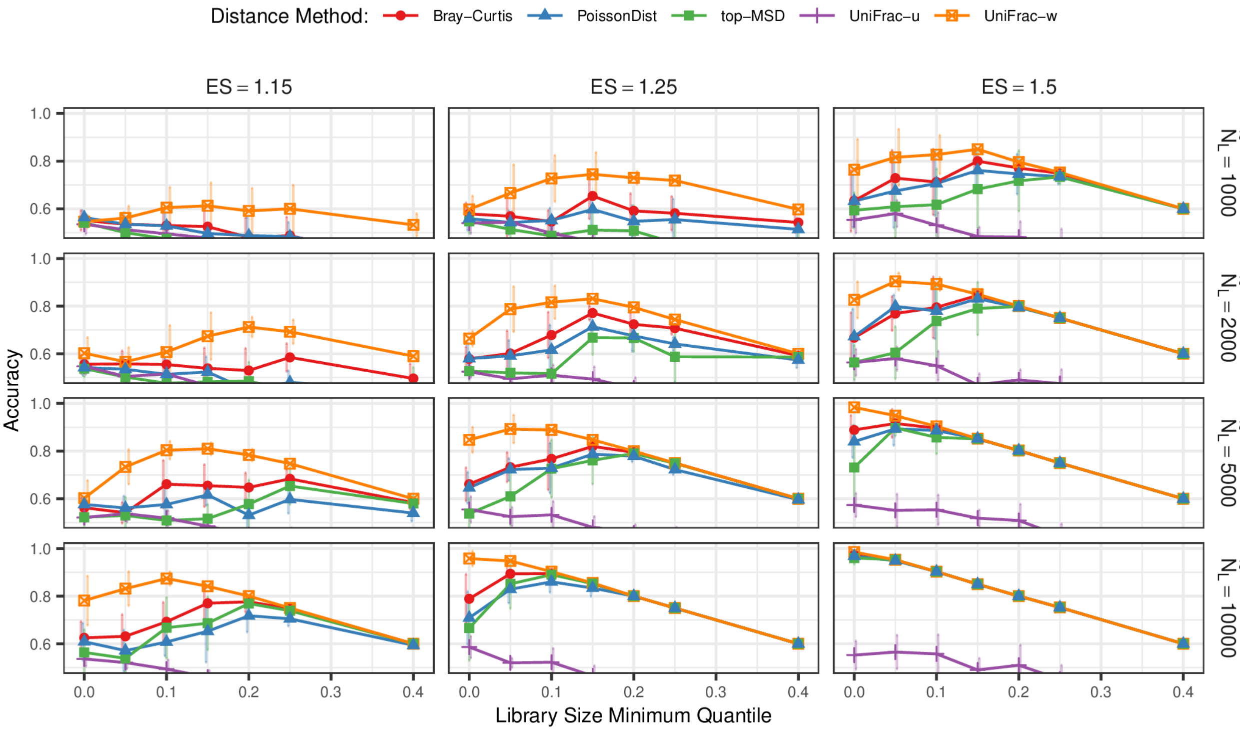

Figure S2. Re-running the R markdown files provided in Protocol S1 of WNWN qualitatively reproduced Figure 5 from WNWN.

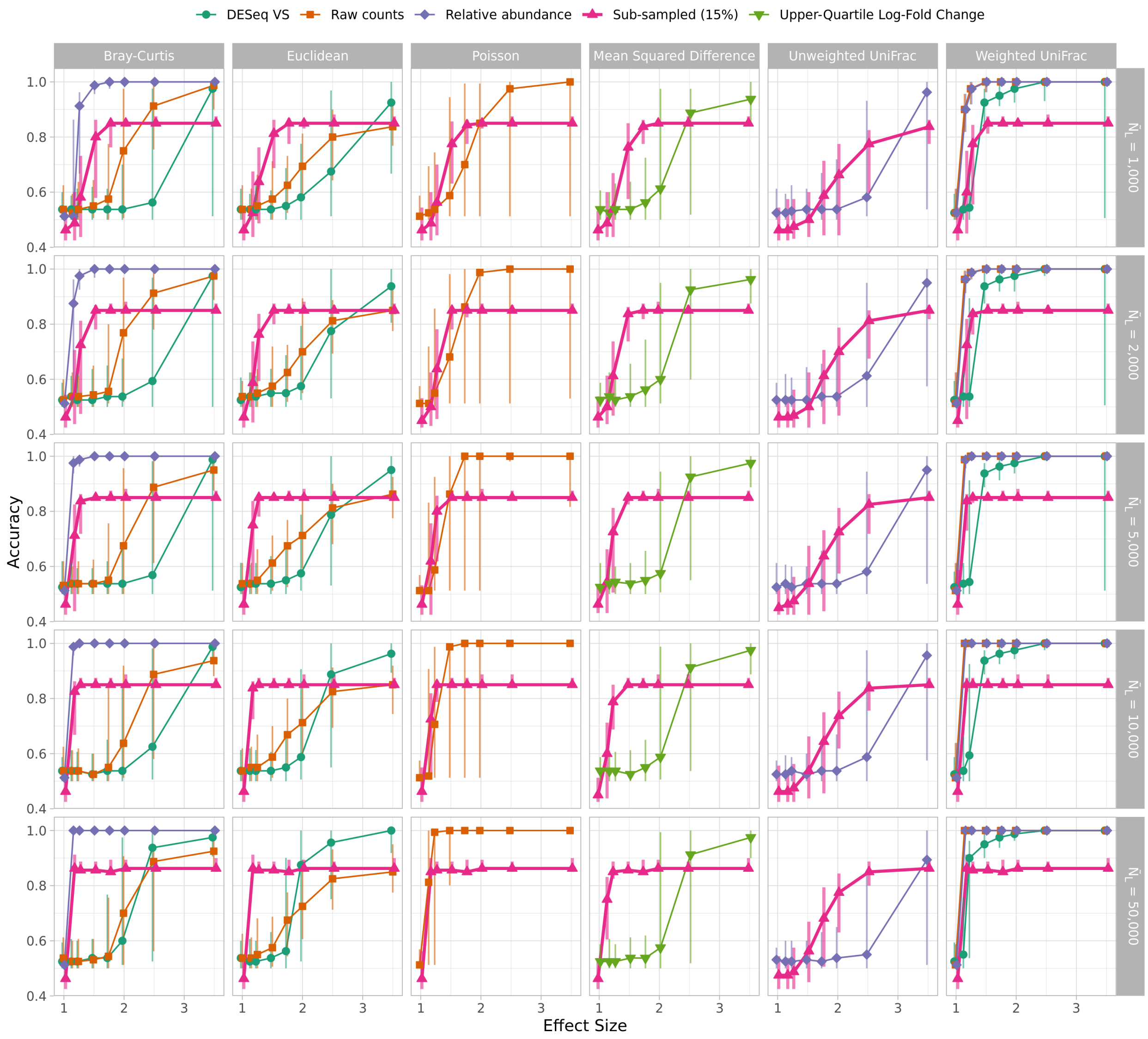

Figure S3. Successful reimplementation and expansion of analysis presented in Figure 4 from WNWN in a Snakemake pipeline. The reimplemented workflow largely borrowed from the original `simulation-cluster-accuracy-server.Rmd` R markdown file provided in WNWN's Protocol S1. The most notable differences include the use of 100 rather than 5 randomizations and the addition of the median sequencing depth ($\tilde{N}_L$) of 50,000. The plotting symbols indicate the median of 100 randomizations and the error bars represent the observed 95% confidence interval. Simulations run at the same effect size are dodged to better reveal overlapping data. The sequencing depths were drawn from the GlobalPatterns dataset and sequences were clustered using PAM.

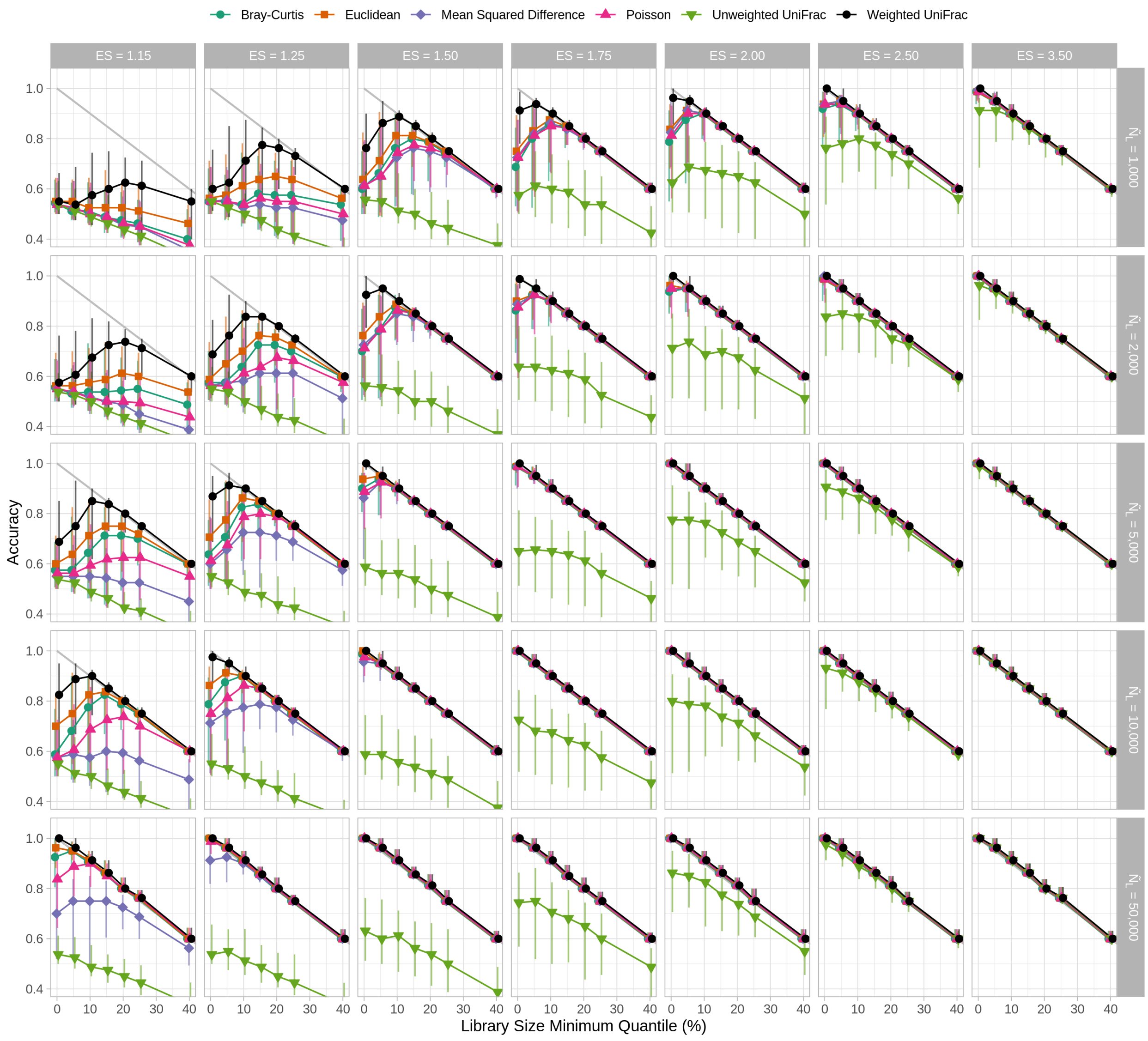

Figure S4. Successful reimplementation and expansion of analysis presented in Figure 5 from WNWN in a Snakemake pipeline. The reimplemented workflow largely borrowed from the original `simulation-cluster-accuracy-server.Rmd` R markdown file provided in WNWN's Protocol S1. The most notable differences include the use of 100 rather than 5 randomizations and the addition of the median sequencing depth ($\tilde{N}_L$) of 50,000. The plotting symbols indicate the median of 100 randomizations and the error bars represent the observed 95% confidence interval. Simulations run at the same effect size are dodged to better reveal overlapping data. A light gray line is shown to indicate the best possible accuracy for each library size minimum quantile value. The sequencing depths were drawn from the GlobalPatterns dataset and sequences were clustered using PAM.

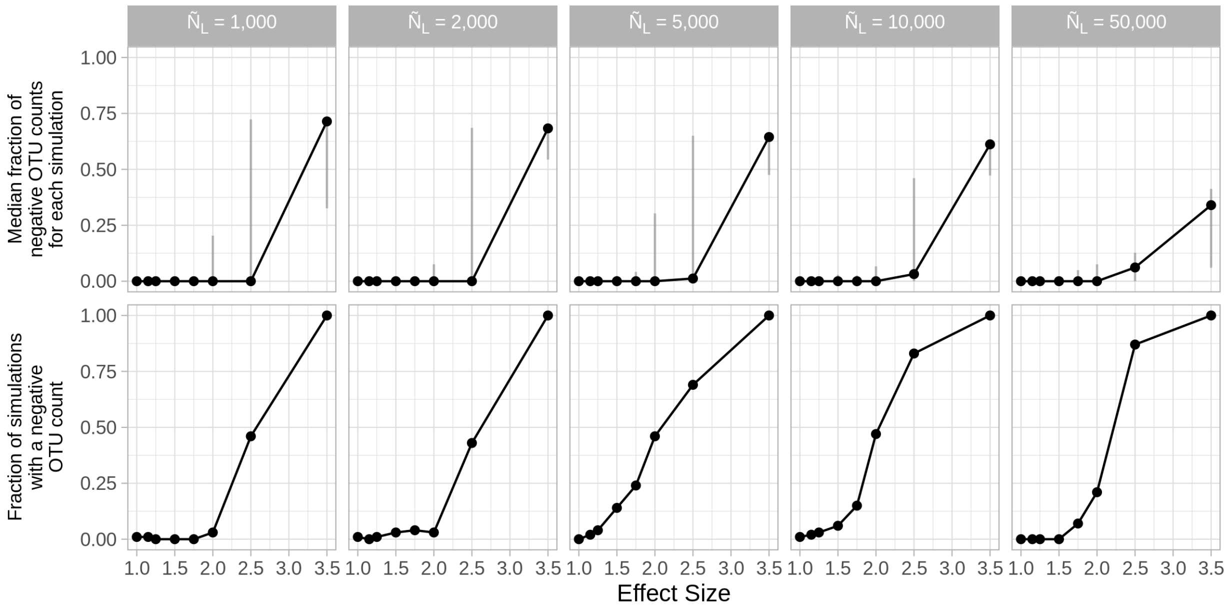

Figure S5. DESeq Variance Stabilization of OTU counts resulted in negative values that were used to calculate Bray-Curtis and Weighted UniFrac distances. The median number of negative OTU counts that had a negative OTU count following normalization increased with the effect size and decreased as $\tilde{N}_L$ increased (first row). The error bars indicate the observe 95% confidence interval. The fraction simulated datasets that had a negative OTU count following normalization also increased with effect size, but increased as $\tilde{N}_L$ increased (second row). For each effect size there were 100 replicate datasets.

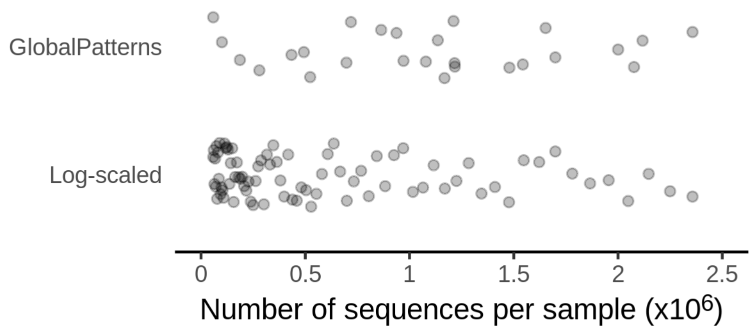

Figure S6. Comparison of the normally distributed sequencing depths from the GlobalPatterns dataset and a log-scaled distribution of sequencing depths. The log-scaled distribution was generated so that each sample in a simulation could have a unique number of sequences and to simulate the skew right distribution commonly seen in microbiome studies.

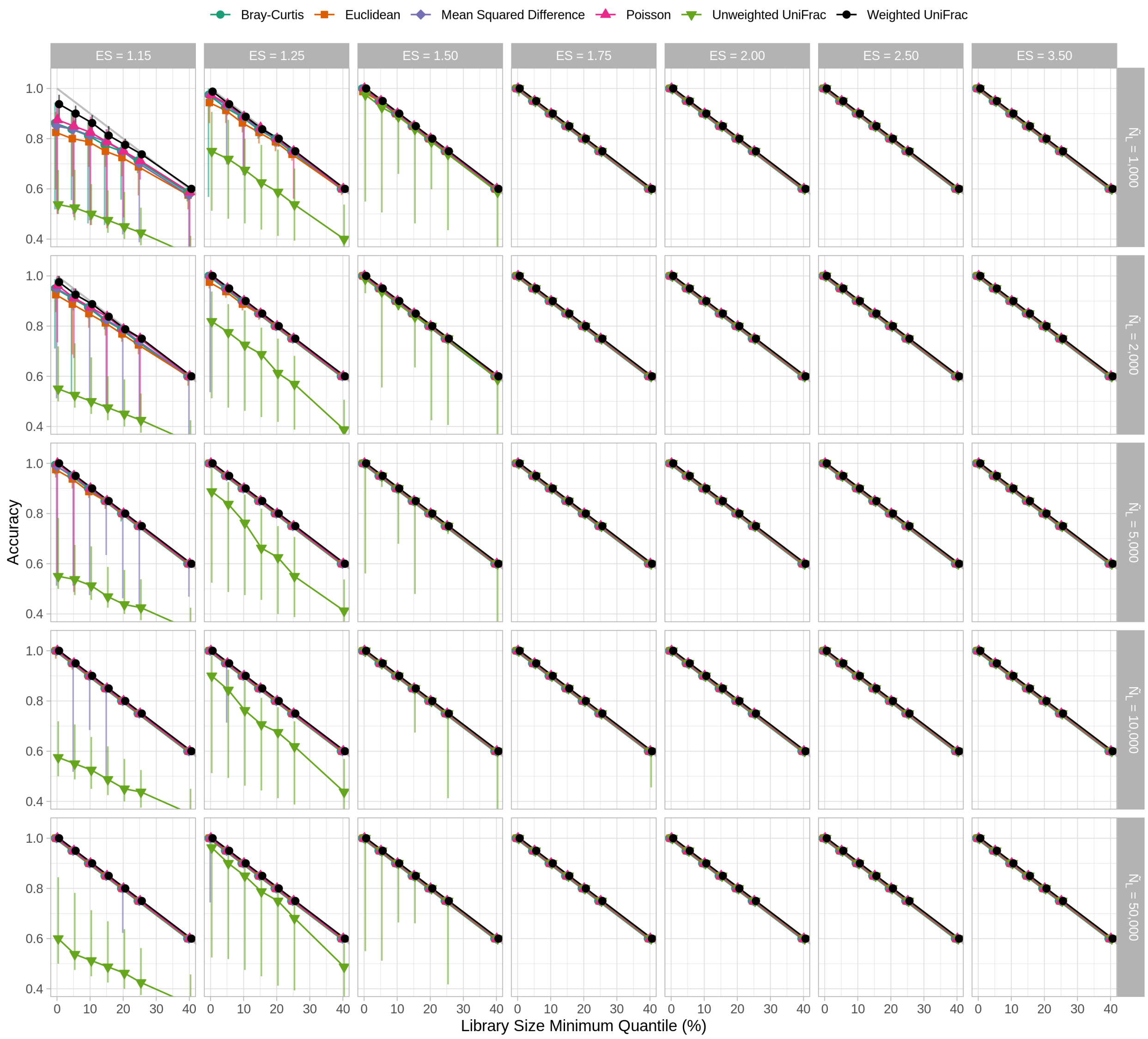

Figure S7. Rarefaction with all samples yielded clustering accuracies that were as good or better than removing the smallest 15% of samples across distance calculation methods. This figure is analogous to Figure 3 except that the sequencing depths for each of the 80 samples in each simulation were drawn without replacement from a log-scaled distribution rather than from the GlobalPatterns sequencing depths.

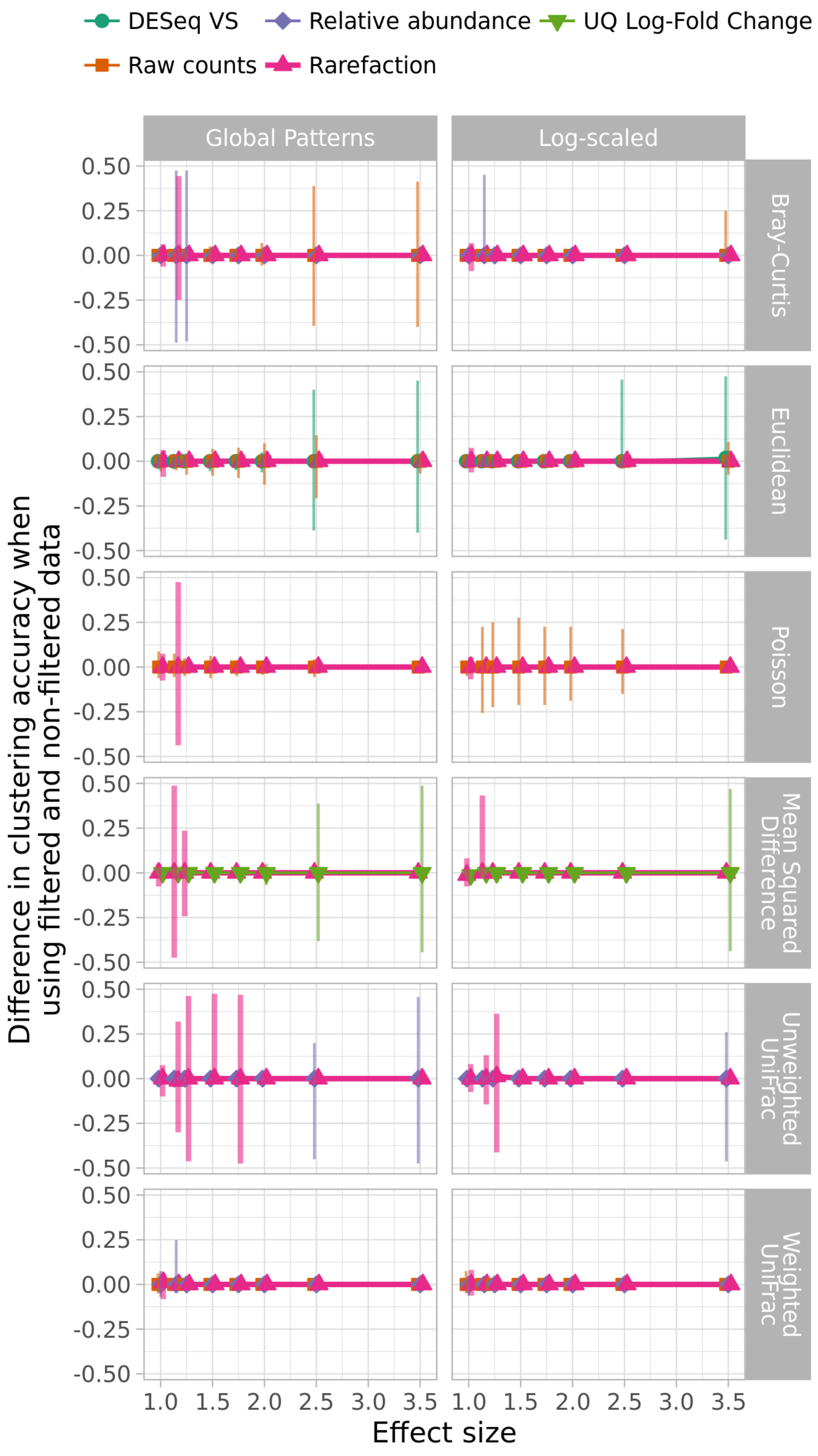

Figure S8. Normalization and distance calculation methods vary in their sensitivity to removal of rare OTUs. Larger values indicate that the clustering accuracy from filtered datasets were larger than those from non-filtered datasets. The median of 100 randomizations did not meaningfully vary from 0.0,

but the observed 95% confidence interval varied considerably. Data are shown for a median sequencing depth ($\tilde{N}_L$) of 10,000 sequences when individual sequencing depths were sampled with replacement from the GlobalPatterns dataset or without replacement from the log-scaled distribution.

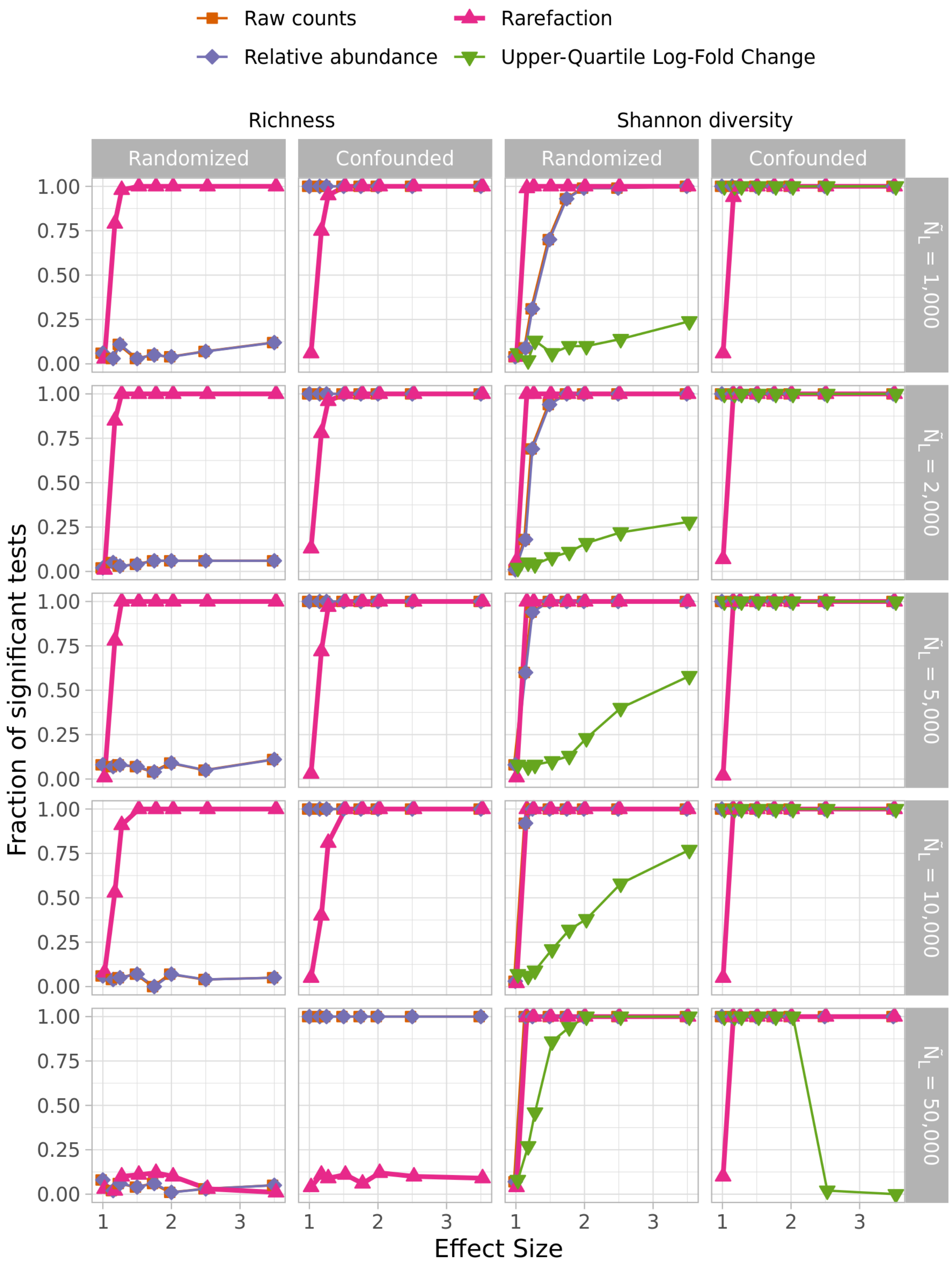

Figure S9. Rarefaction was consistently as good or better than all other normalization methods at controlling for Type I error and maximizing power to detect differences in treatment group using alpha-diversity metrics regardless of whether sequencing depth was confounded by treatment

group when using sequencing depths drawn from a lognormal distribution. Statistical comparisons of OTU richness and Shannon diversity were performed using the non-parametric Wilcoxon two-sampled test. Type I errors were assessed as the fraction of 100 simulations that yielded a significant P-value (i.e., less than or equal to 0.05) at an effect size of 1.00. Power was assessed as the fraction of 100 simulations that yielded a significant P-Value at an effect size of 1.15. Data are shown for when the case when individual sequencing depths were sampled without replacement from the log-scaled distribution.
